# Thiol-ene polymer beads *via* liquid–liquid printing: armored interfaces and photopolymerization *via* graphitic carbon nitride†

**DOI:** 10.1039/d2na00254j

**Published:** 2022-06-23

**Authors:** Cansu Esen, Baris Kumru

**Affiliations:** Max Planck Institute of Colloids and Interfaces, Department of Colloid Chemistry Am Mühlenberg 1 14424 Potsdam Germany baris.kumru@mpikg.mpg.de; Delft University of Technology, Faculty of Aerospace Engineering, Department of Aerospace Structures and Materials Kluyverweg 1 2629 HS Delft Netherlands b.kumru@tudelft.nl

## Abstract

Polymerization of multifunctional thiol-ene molecules is attractive as a proof of concept in photopolymerization, yet the formation of a bead structure is highly restricted. This manuscript will show graphitic carbon nitride based liquid–liquid printing and subsequent photopolymerization to form thiol-ene polymer beads with extreme simplicity and potential scalability.

## Abstract 2

Inspired by artificial caviar formation from molecular cuisine, we can form macroscale thiol-ene polymer beads *via* carbon nitride under static conditions. Carbonization of the so-formed beads yields N-doped gradient carbon spheres.

## Graphical abstract

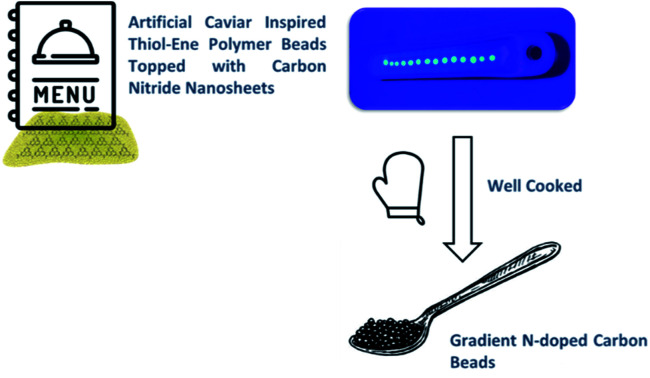

Metal-free graphitic carbon nitride (g-CN) is a visible light active semiconductor and has many appealing applications in photocatalysis.^1–3^ It represents a family of carbon and nitrogen based semiconducting structures with repeating units of triazine, heptazine or tri-*s*-triazine, and all structures are dominated by strong π interactions; hence they are heterogeneous in nature in photocatalytic reactions.^4^ Integration of g-CN into polymer chemistry has been flourishing in the last 4 years and many synthetic examples, from radical polymerization^5–7^ to oxidative polymerization^8,9^ were shown.

g-CN dispersions hold great potential for a wide range of applications from photovoltaics^10,11^ to bioimaging.^12,13^ Once employed in dispersions, g-CN–polymer hybrids can be prepared as well.^14^ While it is possible to encounter aqueous dispersions of g-CN prepared *via* sonication in the literature,^15^ stable organic dispersions of g-CN were achieved once the g-CN surface modification reaction is conducted.^16^ In organic media, the stability arises from electrostatic forces and carbon nitride nanosheets are highly charged.

In recent years, liquid–liquid printing has been popularized due to its versatility to form dimensionally stable soft matter based on interfacial strengthening.^17^ This innovative approach relies on the utilization of oppositely charged molecules (ideally one has to be of long order) in non-miscible phases. Once they are immersed, opposite charges on the interface undergoe a ‘jamming’ effect and a complex liquid shape is stabilized based on solidification of the interface, while the inner phase is still liquid.^18^ Shi and Russell pioneered many possible charged molecules in alternative solvents to produce complex architectures.^19–23^ Our group has investigated the potential of organic g-CN (denoted as CMp-vTA) as an interface stabilizer to access liquid–liquid printing and interfacial photoactivity of an edible oil-in-water printed soft structure by photocatalytic dye degradation was exhibited.^24^ Overall, liquid–liquid printing is a nanoarchitectonics concept^25^ as oppositely charged natural molecules can form biological structures in nature.^26^

In this communication, we will take a step further in g-CN based liquid–liquid printing and photoactivity will be harnessed to form solid polymer beads (Scheme 1).

**Scheme 1:**
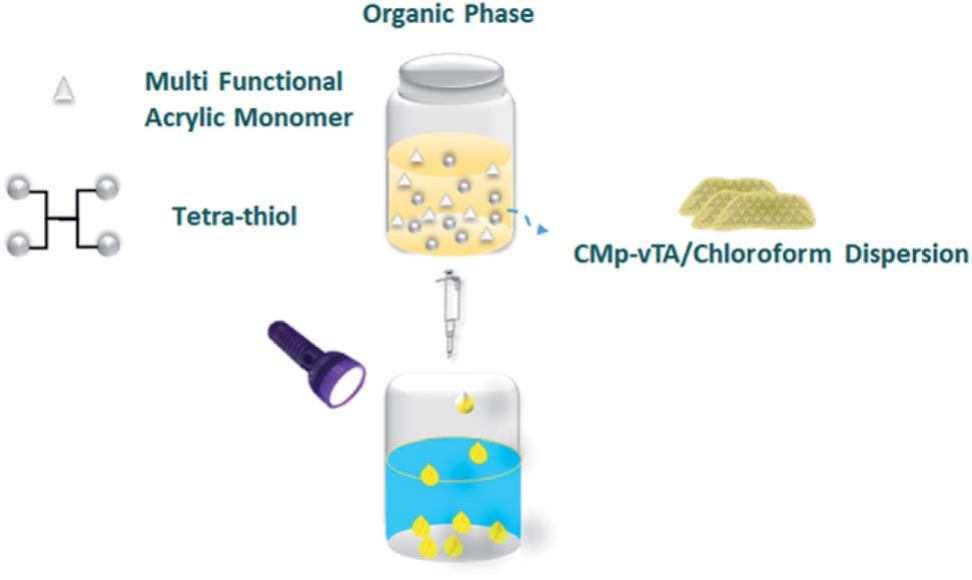
Fabrication of thiol-ene polymer beads *via* the liquid–liquid printing technique.

Polymerization of multifunctional thiol and ene molecules is shown as a proof of concept in photopolymerization and 3D printing.^27,28^ While it is possible to form many appealing macroscale thiol-ene geometries *via* 3D printing, heterophase polymerization of thiol-ene is scarce.^29^ It is quite challenging to conduct traditional suspension polymerization on such systems, and micron-sized particle fabrication is possible *via* microfluidics.^30^ Here we will use interfacial nanoscale interactions to form macroscale thiol-ene polymer beads *via* liquid–liquid printing. A CMp-vTA dispersion in chloroform is prepared and mixed with pentaerythritol tetraacrylate and tetrakis(3-mercaptopropionate) (TT bead, 1 : 1 molar eq.). We also alternated the vinyl monomer library by employing 1,3,5-triallyl-1,3,5-triazine-2,4,6(1*H*,3*H*,5*H*)-trione (TTA bead) and 2,4,6-triallyloxy-1,3,5-triazine (TTO bead, Scheme S1†). A boat shaped aluminium crucible is filled with boric acid aqueous solution. The organic phase is dropped into aqueous solution and stable liquid bead formation is observed due to the interaction of CMp-vTA sheets with boric acid (Fig. 1a inlet and ESI Video 1†). Under UV light, CMp-vTA initiates polymerization and within 2 hours organic macroscale droplets solidify. This process can be automated and is highly energy efficient as no heat or stirring is required. It is optimized to form beads within the range of 1 mm to 1 cm. It is important to underline that the system worked best on aluminium surfaces, and glass or polymer surfaces gave defective structures. After purification, ICP was conducted to ensure that boron is removed from the polymer bead surface and indeed almost no boron was observed (Table S1†).

**Fig. 1:**
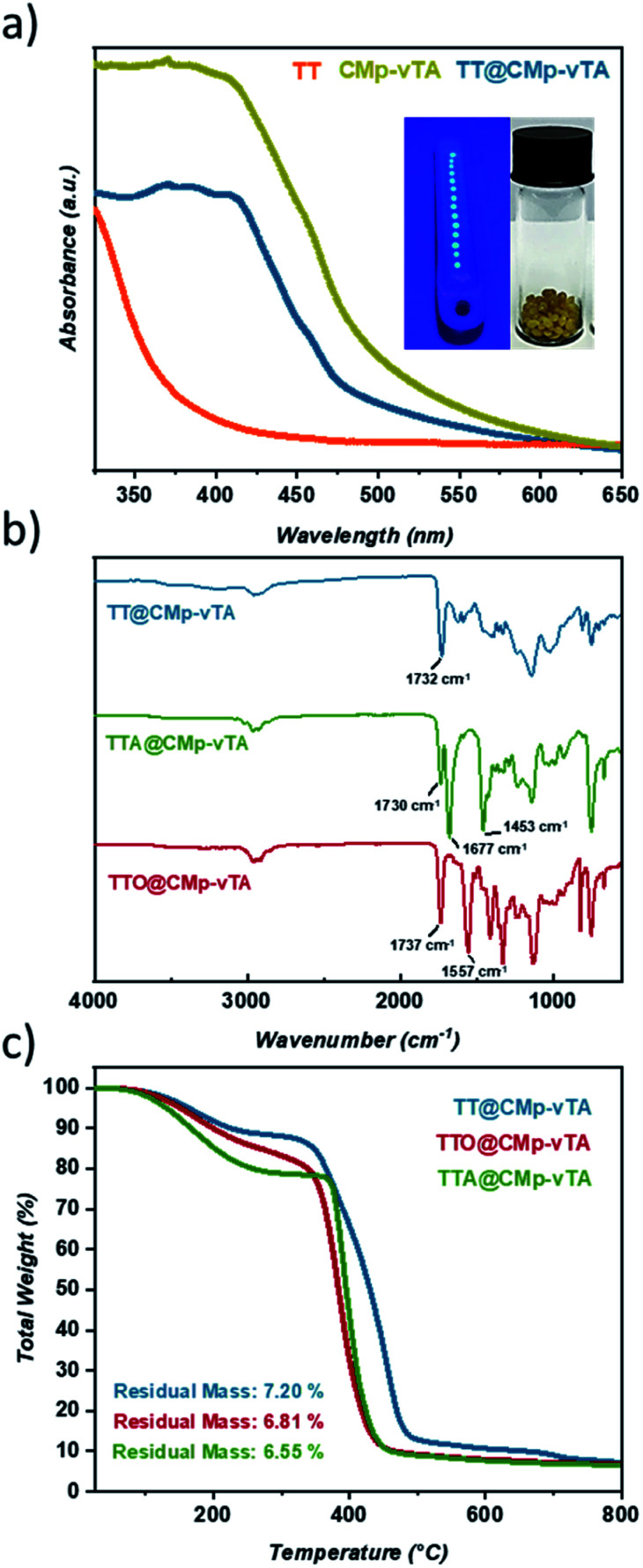
Solid UV-Vis spectra of TT, TT@CMp-vTA and pristine CMp-vTA powder along with digital images of TT@CMp-vTA during synthesis (left) and after purification (right) (a), FT-IR spectra of TT@CMp-vTA, TTO@CMp-vTA, and TTA@CMp-vTA (b), and TGA measurement of TT@CMp-vTA, TTO@CMp-vTA, and TTA@CMp-vTA (c).

Solid UV-Vis spectra of TT and TT@CMp-vTA exhibit a significant absorption difference whilst the characteristic broad spectrum of pristine CMp-vTA (350–450 nm) matches with that of TT@CMp-vTA that can be attributed to CMp-vTA integration (Fig. 1a). Structural investigation performed *via* FT-IR analysis simply confirmed varied thiol-ene bead compositions. The ester C=O stretching modes that appeared at 1732 cm^−1^ for the TT@CMp-vTA sample differed from those for the TTA@CMp-vTA sample regarding its isocyanurate based structure; C=O stretching at 1730 cm^−1^, N–C=O 1677 cm^−1^, and aromatic C–N stretching at 1453 cm^−1^. TTO@CMp-vTA spectra displayed intense cyanurate absorption bands at 1737 cm^−1^, 1557 cm^−1^ and roughly until 1330 cm^−1^ corresponding to N=C–O and aromatic ring vibrations of C–N and C=N bonds, respectively. The overall differences in all spectra in the lower fingerprint region additionally confirm the structural variation of the resulting thiol-ene beads (Fig. 1b).

Thermogravimetric profiles demonstrated a slight difference in regard to the thermal stability of samples including the ones that do not possess CMp-vTA. Regardless of CMp-vTA incorporation, significant mass loss starts at around 350 °C and eventuates closely at 435 °C for TTA, TTO, TTA@CMp-vTA, and TTO@CMp-vTA samples unlike TT and TT@CMp-vTA beads that are more stable up to 485 °C. Regarding residual mass amounts, which indicated possible thermal condensation at high temperature (up to 800 °C) that might lead to carbonization of polymer samples, they are investigated and discussed further in the following parts (Fig. 1c and S1†).

The scanning electron microscopy (SEM) image of TT@CMp-vTA exhibited very smooth surface morphology that underlines the favoured colloidally stable (interfacial jamming) synthetic condition during polymerization (Fig. 2a and S3a1, a2†). Besides, the confocal laser scanning microscopy image (cross section of half the TT@CMp-vTA bead) simply confirmed the surface-restricted location of CMp-vTA based on intense green luminescence around 540 nm as it is a characteristic emissive property of CMp-vTA (Fig. 2b). Furthermore, EDX mapping revealed a homogeneous distribution of elements without any compartmentalization (Fig. 2c).

**Fig. 2:**
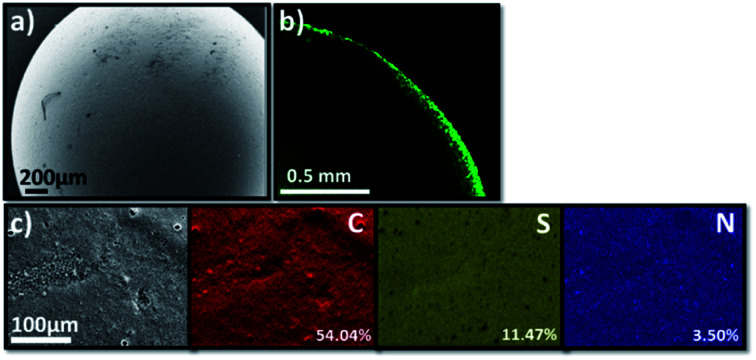
Scanning electron microscopy image of TT@CMp-vTA (a), the cross sectional confocal laser scanning microscopy image of a half-cut TT@CMp-vTA bead (b), and elemental mapping of TT@CMp-vTA *via* EDX (c).

In order to investigate the solvent uptake performance of TT@CMp-vTA beads, various solvents were used and according to the results, the highest solvent uptake ratio was detected in dipolar aprotic acetone (46.8%) and polar aprotic THF (32%) followed by polar protic ethanol (29.5%). Beads in nonpolar solvents such as toluene (1.1%) and hexane (1.5%) or polar water (0.7%) did not show efficient uptake (Fig. S2†).

As CMp-vTA particles reside on the surface with no porosity, significant photoactivity is not observed as it takes place on porous interfaces in g-CN hybrids.^31^ As expected, our studies on photocatalytic RhB degradation *via* TT@CMp-vTA beads showed no remarkable activity on RhB degradation (Fig. S3†), which underlines the restricted photoactivity of TT@CMp-vTA beads. This observation is in good agreement with general knowledge that porosity is needed for (photo)catalysis on hybrid structures where the photoactive material should be at the inner porous interface and not on the surface.^32^ As the mechanism of liquid–liquid printing solely relies on interface interactions (Scheme S2†), all CMp-vTA particles from the organic phase rush towards the interface to accommodate stable structure formation.

In other words, the beads obtained in this study seem covalently coated with CMp-vTA particles. Possessing a thermally stable outer layer is highly interesting for polymer carbonization (‘compartmentalized microchamber effect’). In recent years, carbonization of polymeric materials has been a growing trend to manufacture doped carbonaceous matter for electrochemical and battery applications.^33–35^ While many polymers reach ceiling temperature and yield no carbon, polymers yielding special carbons require complicated synthesis and overall very expensive materials to be employed. Additionally, it is complicated to have monolithic doped carbon structures except in exceptional cases.^36–39^ To examine our case, carbonization of the so-formed beads was conducted. To begin with, the reduced bead size of TT@CMp-vTA beads (carbonized analogue is labeled CTT@CMp-vTA) can be clearly seen *via* scanning electron microscopy images (Fig. 3a and S4a, b†). The preserved bead shape (in a range of 0.5–4 mm) after carbonization is also proven visually *via* digital images (Fig. 3b), that is unique for TT@CMp-vTA beads since the rest of the beads (TTA@CMp-vTA and TTO@CMp-vTA) are obtained in powder form with metallic black color (named CTTA@CMp-vTA and CTTO@CMp-vTA, respectively). EDX mapping exhibits the abundance of atoms in the CTT@CMp-vTA sample; an increased C content (wt%) and a significant decrease in the S atom (wt%) compared to TT@CMp-vTA were noted (Fig. 3c). Combustive elemental analysis of all carbonized samples showed a high carbon content with N and S codoping (Fig. 3d and Table S2†). For CTT@CMp-vTA, we believe that the reason for the notable difference in the detected N content *via* EDX (12.26%) and combustive elemental analysis (3.22%) arises from the gradient structure. CMp-vTA, a nitrogen rich compound, was exclusively located on the surface of precursor beads. Thus EDX highlights the high N content on the surface of the carbonized analogue, whereas elemental analysis investigates the whole sample and thus the N content decreases in the inner parts. One can argue the formation of core–shell like N-content gradient carbon beads in this case.

**Fig. 3:**
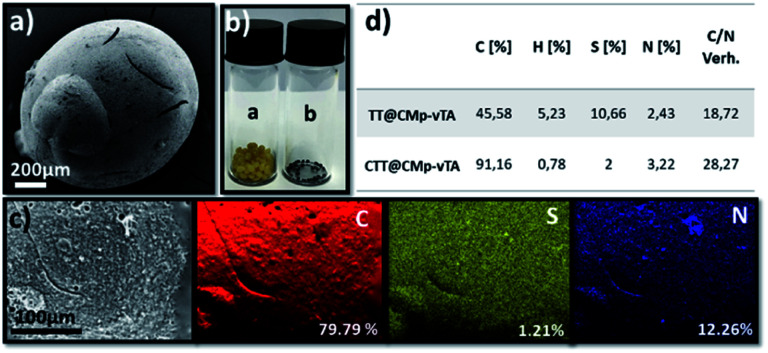
Scanning electron microscopy image of CTT@CMp-vTA (a), digital images of TT@CMp-vTA and CTT@CMp-vTA (a) and (b) (b), elemental mapping of CTT@CMp-vTA *via* EDX (c), and the combustive elemental analysis results of TT@CMp-vTA and CTT@CMp-vTA (d).

Furthermore, XRD profiles of all carbonized samples exhibited broad diffractions at 25.2° and 43.75° that can be assigned to (002) and (100) planes of the amorphous carbon structure (Fig. S5†). Moreover, Raman spectra of carbonized CMp-vTA containing samples showed the characteristic defect-induced band (D band, 1358 cm^−1^) and crystalline band (G band, 1585 cm^−1^) with varied *I*_D_/*I*_G_ (intensity ratio of D to G band) values. The CTT@CMp-vTA sample, which is the only carbon sample preserved in bead shape, exhibited the lowest *I*_D_/*I*_G_ value which can be considered as having a higher ordering degree than the CTTA@CMp-vTA and CTTO@CMp-vTA samples that are obtained in powder form after carbonization (Fig. S6†). It is important to underline that almost no boron was detected in carbonized structures as well (Table S1†).

According to the N_2_ sorption results, CMp-vTA based carbonized samples (CTT@CMp-vTA, CTTA@CMp-vTA, and CTTO@CMp-vTA) possess very low surface area (∼20 m^2^ g^−1^) as neither a porogen nor a template was applied during synthesis (Fig. S7†). Enhancing the surface area and elucidating electrochemical applications open the door for further studies.

Overall, a simple synthetic methodology prone to being automated to attain thiol-ene polymer beads can be followed by carbonization to obtain scalable S, N-codoped carbon materials from very cheap and available monomers that would be highly interesting for electrocatalysis and batteries.

## Conclusions

Liquid–liquid printing has been an attractive method to manufacture soft matter with high structural complexity based on the interfacial jamming effect of oppositely charged molecules on different phases. Graphitic carbon nitride particles in the organic phase can reinforce the interface when injected into aqueous solutions. Once the organic phase contains polymerizable multifunctional thiol and -ene compounds, light irradiation triggers polymerization thanks to the semiconducting properties of carbon nitride particles. In this manuscript, we formed crosslinked thiol-ene polymer beads with extreme simplicity and scalability that can potentially be automated (for example with an artificial caviar producing device from molecular gastronomy). As expected, the resulting macroscale beads were not photoactive as inner porosity is hindered by carbon nitride particles on the surface. However; this innovative, simple and cheap design based on the nanoarchitectonics principle provided sulfur and nitrogen-codoped carbon materials upon carbonization, which is a new avenue in the carbonization of polymeric materials. We were able to obtain monolithic S and N co-doped carbon beads with a core–shell N-gradient architecture that would be extremely attractive for carbocatalysis.

## Experimental

### Materials

1,3,5-Triallyl-1,3,5-triazine-2,4,6(1*H*,3*H*,5*H*)-trione (98%, Sigma Aldrich), 2,4,6-triallyloxy-1,3,5-triazine (97%, Sigma Aldrich), 2,4,6-trimethylbenzoyl diphenylphosphine oxide (DPO, 98%, Sigma Aldrich), 2,4-diamino-6-phenyl-1,3,5 triazine (Mp, 97%, Sigma Aldrich), 4-methyl-5-vinylthiazole (vTA, 97%, Sigma Aldrich), cyanuric acid (C, 98%, Sigma Aldrich), acetone (ACS reagent, ≥99.5%, Sigma Aldrich), boric acid (ACS reagent, ≥99.5%, Sigma Aldrich), chloroform (HPLC, ≥99.8%, contains 0.5–1.0% ethanol as the stabilizer, Sigma Aldrich), ethanol (HPLC, ≥99.8%, Sigma Aldrich), pentaerythritol tetrakis(3-mercaptopropionate) (>95%, Sigma Aldrich), pentaerythritol tetraacrylate (contains 350 ppm monomethyl ether hydroquinone as an inhibitor, Sigma Aldrich), rhodamine B (RhB, 95%, Sigma Aldrich), tetrahydrofuran (THF, anhydrous 99.9%, HPLC grade, Sigma Aldrich), and toluene (anhydrous 99.8%, Sigma Aldrich) were used. Ceramic crucibles for bead synthesis (boat shape 97 × 17 × 12 mm) were obtained from VWR. Ultraviolet (UV) light irradiation was performed *via* a 30 W UV chip (Fdit 395–400 Nm UV LED) connected to a self-made circuit and a cooling system. Visible light irradiation was implemented *via* two 50 W LEDs (Chip Bulb Light DIY White 3800LM 6500 K) connected to a self-made circuit and a cooling system.

### Characterization

A CMp-vTA/chloroform suspension was prepared in a sonication bath at 50% amplitude from Elma (Transsonic T310). Solid-state ultraviolet-visible (UV-Vis) spectroscopy for ground samples was performed *via* a Cary 500 Scan spectrophotometer equipped with an integrating sphere. Fourier transform infrared (FT-IR) spectra were acquired on a Nicolet iS 5 FT-IR spectrometer. Thermogravimetric analysis (TGA) was performed *via* a Thermo Microbalance TG 209 F1 Libra (Netzsch, Selb, Germany) under a nitrogen atmosphere with a heating rate of 10 K min^−1^ by using aluminum crucibles for samples. Scanning electron microscopy (SEM) and EDX elemental mapping were performed using a JSM-7500F (JEOL) microscope equipped with an Oxford Instruments X-Max 80 mm^2^ detector for the determination of both elemental composition and morphology. A fluorescence image of TT@CMp-Vta was obtained by confocal laser scanning microscopy (CLSM, TCS SP5, Leica, Germany). Combustive elemental analysis was recorded *via* a Vario Micro device. X-ray diffraction (XRD) patterns of finely ground samples were obtained by using a Bruker D8 Advance X-ray diffractometer Cu-Kα (*λ* = 0.154 nm) equipped with a NaI scintillation counter-Scinti-Detector (diffraction pattern was recorded in the 2*θ* range between 4 and 70° with steps of 0.05° and acquisition time of 2 s per step). Trace analysis of boron was performed *via* inductively coupled plasma optical emission spectroscopy (ICP-OES Optima 8000). Raman spectra were recorded using a confocal Raman microscope alpha300 (WITec, Germany) coupled with laser excitation at a wavelength of 532 nm. N_2_ sorption measurements were accomplished with N_2_ at −196 °C, after degassing the sample at 150 °C for 20 hours under vacuum, using a Quantachrome Quadrasorb SI porosimeter. The specific surface area was calculated by applying the Brunauer–Emmet–Teller model in the relative pressure region of (0–0.05) for the adsorption branch (ABET).

Solvent uptakes were calculated manually. 40 mg (*W*_d_) purified TT@CMp-vTA beads were weighed into separate vials for various solvent additions (2 mL of acetone, ethanol, water, toluene, and THF) and then the vials were capped and left for 24 hours at room temperature. Beads treated with solvents were weighed separately (*W*_s_) and solvent uptake was calculated by using the following formula for each solvent type:Solvent uptake = (*W*_s_ − *W*_d_)/*W*_d_ × 100%

### Synthesis of phenyl doped g-CN (CMp)

Phenyl-modified g-CN (CMp) was synthesized according to the literature.^40^ 1.3 g of cyanuric acid and 1.8 g of 2,4-diamino-6-phenyl-1,3,5-triazine were weighed and mixed with 50 mL distilled water and shaken overnight. After centrifugation at 5000 rpm for 5 minutes, the precipitate was dried at 60 °C under vacuum overnight. The dried product is transferred into a capped crucible and placed into a N_2_ protected oven at 450 °C for 2 hours, with a heating rate of 2.3 °C min^−1^. After cooling to ambient temperature, yellow CMp powder was obtained and well ground prior to use.

### Synthesis of CMp-vTA

CMp-vTA particles were synthesized according to the cited literature.^16^ 100 mg CMp was mixed with 1 mL vTA and sonicated for 5 minutes in a sonic bath. The mixture was degassed with nitrogen flux for 10 minutes and placed between 2 50 W LEDs and stirred for 3 hours under continuous visible light irradiation, and for purification, the mixture was filtered and washed with ethanol 3 times (20 mL each portion) and dried under vacuum at 60 °C overnight. After cooling to ambient temperature, dark-yellow vTA-CMp powder was obtained and well ground before usage.

### Preparation of the CMp-vTA/chloroform dispersion

40 mg vTA-CMp was sonicated in 1 mL of chloroform in a sonic bath 3 times/30 minutes cycles to exfoliate carbon nitride. The dispersion was set to rest for 1 hour prior to use in order to allow the sedimentation of larger particles.

### Synthesis of CMp-vTA incorporated thiol-ene polymer beads (TT@CMp-vTA, TTA@CMp-vTA, and TTO@CMp-vTA)

Pentaerythritol tetraacrylate and tetrakis(3-mercaptopropionate) (1 : 1 eq.) were weighed in a glass vial, 1 mL of the freshly prepared CMp-vTA/chloroform dispersion was added into the vial and all components were mixed properly. Meanwhile, boat shape crucibles were filled with aqueous boric acid solution (4 wt%). The as-prepared monomer and CMp-vTA containing the organic mixture were carefully dropped into boric acid solution as demonstrated in a digital video reported in the ESI.† Subsequently, the crucibles were placed under a UV light source at a distance of 15 cm from the top to the crucible level, for 2 hours to complete polymerization. Afterwards, the as-prepared polymer beads were washed with an adequate amount of THF and water three times and left in a fume hood for drying (denoted as TT@CMp-vTA). In order to vary the bead composition, the same process was performed by conducting 1,3,5-triallyl-1,3,5-triazine-2,4,6(1*H*,3*H*,5*H*)-trione and 2,4,6-triallyloxy-1,3,5-triazine with tetrakis(3-mercaptopropionate) (1 : 1 eq.) and the resulting samples were denoted as TTA@CMp-vTA and TTO@CMp-vTA, respectively.

Given the fact that CMp-vTA is the photoinitiator for photopolymerization and an interface stabilizer to provide bead shape, reference materials in the absence of CMp-vTA were synthesized in bulk by the addition of DPO (1 wt%) and the obtained materials were labeled as TT, TTA, and TTO in accordance with CMp-vTA containing analogues.

### Carbonization of CMp-vTA incorporated thiol-ene beads

After the purification & drying steps, beads were separately placed in capped crucibles and placed into a N_2_ protected chamber furnace at 800 °C for 30 minutes with a heating rate of 5 °C min^−1^. The resulting materials were named according to source beads *e.g.* CTT@CMp-vTA were derived from TT@CMp-vTA (CTTA@CMp-vTA made by TTA@CMp-vTA and CTTO@CMp-vTA made by TTO@CMp-vTA).

### Photocatalytic RhB degradation

The photocatalytic activity of TT@CMp-vTA beads was investigated *via* photocatalytic degradation of aqueous RhB solution (50 mg bead: 2 mL of 2 ppm RhB in deionized water) under visible light irradiation. Beads were mixed with RhB dye solution in a glass vial in the dark at continuous stirring for 30 minutes in order to achieve an adsorption–desorption equilibrium. Afterwards, the irradiation was performed using a white 50 W LED (Chip Bulb Light DIY White 3800LM 6500 K) under continuous stirring for 9 hours. Degradation performance was followed *via* UV-Vis spectroscopy by taking conduction samples every 3 hours.

## Conflicts of interest

There are no conflicts to declare.

## Supplementary Material

NA-004-D2NA00254J-s001

NA-004-D2NA00254J-s002

## References

[cit1] Mazzanti S., Manfredi G., Barker A. J., Antonietti M., Savateev A., Giusto P. (2021). ACS Catal..

[cit2] Schlomberg H., Kröger J., Savasci G. k., Terban M. W., Bette S., Moudrakovski I., Duppel V., Podjaski F., Siegel R. e., Senker J. r., Dinnebier R. E., Ochsenfeld C., Lotsch B. V. (2019). Chem. Mater..

[cit3] Han Q., Wang B., Zhao Y., Hu C., Qu L. (2015). Angew. Chem., Int. Ed..

[cit4] Xiong W., Huang F., zhang R.-Q. (2020). Sustainable Energy Fuels.

[cit5] Gaddam S. K., Pothu R., Boddula R. (2020). Polym. Compos..

[cit6] Zhang L., Ye G., Huo X., Xu S., Chen J., Matyjaszewski K. (2019). ACS Omega.

[cit7] Kaya K., Kiskan B., Kumru B., Schmidt B. V. K. J., Yagci Y. (2020). Eur. Polym. J..

[cit8] Esen C., Antonietti M., Kumru B. (2021). ChemPhotoChem.

[cit9] Kumru B., Cruz D., Heil T., Antonietti M. (2020). Chem. Mater..

[cit10] Chen X., Liu Q., Wu Q., Du P., Zhu J., Dai S., Yang S. (2016). Adv. Funct. Mater..

[cit11] Zhou L., Xu Y., Yu W., Guo X., Yu S., Zhang J., Li C. (2016). J. Mater. Chem. A.

[cit12] Zhuang Q., Guo P., Zheng S., Lin Q., Lin Y., Wang Y., Ni Y. (2018). Talanta.

[cit13] Kim J. K., Park S., Yoo R. J., Jeong H. J., Oh J., Lee Y. J., Park S., Kim D. W. (2018). Chem.–Eur. J..

[cit14] Yandrapalli N., Robinson T., Antonietti M., Kumru B. (2020). Small.

[cit15] Xu J., Shalom M. (2019). ChemPhotoChem.

[cit16] Kumru B., Cruz D., Heil T., Schmidt B. V. K. J., Antonietti M. (2018). J. Am. Chem. Soc..

[cit17] Huang C., Sun Z., Cui M., Liu F., Helms B. A., Russell T. P. (2016). Adv. Mater..

[cit18] Shi S., Liu X., Wu X., Wang D., Forth J., Russell T. P. (2018). Adv. Mater..

[cit19] Qian B., Shi S., Wang H., Russell T. P. (2020). ACS Appl. Mater. Interfaces.

[cit20] Gu P.-Y., Chai Y., Hou H., Xie G., Jiang Y., Xu Q.-F., Liu F., Ashby P. D., Lu J.-M., Russell T. P. (2019). Angew. Chem., Int. Ed..

[cit21] Yin Y., Liu T., Wang B., Yin B., Yang Y., Russell T. P., Shi S. (2022). Adv. Funct. Mater..

[cit22] Sun H., Li M., Li L., Liu T., Luo Y., Russell T. P., Shi S. (2021). J. Am. Chem. Soc..

[cit23] Sun H., Li L., Russell T. P., Shi S. (2020). J. Am. Chem. Soc..

[cit24] Cao Q., Amini S., Kumru B., Schmidt B. V. K. J. (2021). ACS Appl. Mater. Interfaces.

[cit25] Azzaroni O., Ariga K. (2019). Mol. Syst. Des. Eng..

[cit26] Zhao Q., Lee D. W., Ahn B. K., Seo S., Kaufman Y., Israelchvili J. N., Waite J. H. (2016). Nat. Mater..

[cit27] Xiao P., Dumur F., Graff B., Fouassier J. P., Gigmes D., Lalevee J. (2013). Macromolecules.

[cit28] Cook C. C., Fong E. J., Schwartz J. J., Porcincula D. H., Kaczmarek A. C., Oakdale J. S., Moran B. D., Champley K. M., Rackson C. M., Muralidharan A., McLeod R. R., Shusteff M. (2020). Adv. Mater..

[cit29] Cobb J. S., Chapusha C., Gaikwad J., Michael J., Janorkar A. V. (2021). Mater. Adv..

[cit30] Prasath R. A., Gokmen M. T., Espeel P., Prez F. E. D. (2010). Polym. Chem..

[cit31] Esen C., Antonietti M., Kumru B. (2021). J. Appl. Polym. Sci..

[cit32] Cao Q., Barrio J., Antonietti M., Kumru B., Shalom M., Schmidt B. V. K. J. (2020). ACS Appl. Polym. Mater..

[cit33] Sharma S. (2019). Materials.

[cit34] Wang H., Shao Y., Mei S., Lu Y., Zhang M., Sun J.-k., Matyjaszewski K., Antonietti M., Yuan J. (2020). Chem. Rev..

[cit35] Yan L., Han D., Xiao M., Ren S., Li Y., Wang S., Meng Y. (2017). J. Mater. Chem. A.

[cit36] Brandi F., Baumel M., Molinari V., Shekova I., Lauermann I., Heil T., Antonietti M., Al-Naji M. (2020). Green Chem..

[cit37] Liu B., Shi R., Ma X., Chen R., Zhou K., Xu X., Sheng P., Zeng Z., Li L. (2021). Carbon.

[cit38] Ma X., Zou B., Cao M., Chen S.-L., Hu C. (2014). J. Mater.
Chem. A.

[cit39] Geng Z., Xiao Q., Lv H., Li B., Wu H., Lu Y., Zhang C. (2016). Sci. Rep..

[cit40] Cui Q., Xu J., Wang X., Li L., Antonietti M., Shalom M. (2016). Angew. Chem., Int. Ed..

