# Supplementary material for: Thiol-ene polymer beads via liquid–liquid printing: armored interfaces and photopolymerization via graphitic carbon nitride
Source: Nanoscale Adv. 2022 Jun 23;4(15):3136–41. doi: 10.1039/d2na00254j (PMC9418565; doi:10.1039/d2na00254j)
Supplement: NA-004-D2NA00254J-s001 [file NA-004-D2NA00254J-s001.pdf]

Electronic Supplementary Material (ESI) for Nanoscale Advances.
This journal is © The Royal Society of Chemistry 2022

Supporting Information for

Thiol-ene Polymer Beads via Liquid-Liquid Printing: Armored Interfaces and Photopolymerization via Graphitic Carbon Nitride

Cansu Esen[a], Baris Kumru[a,b,*]

[a.] *Max Planck Institute of Colloids and Interfaces, Department of Colloid Chemistry, Am Mühlenberg 1, 14424 Potsdam, Germany.*
[b.] *Delft University of Technology, Faculty of Aerospace Engineering, Department of Aerospace Structures and Materials, Kluyverweg 1, 2629 HS Delft, the Netherlands.*

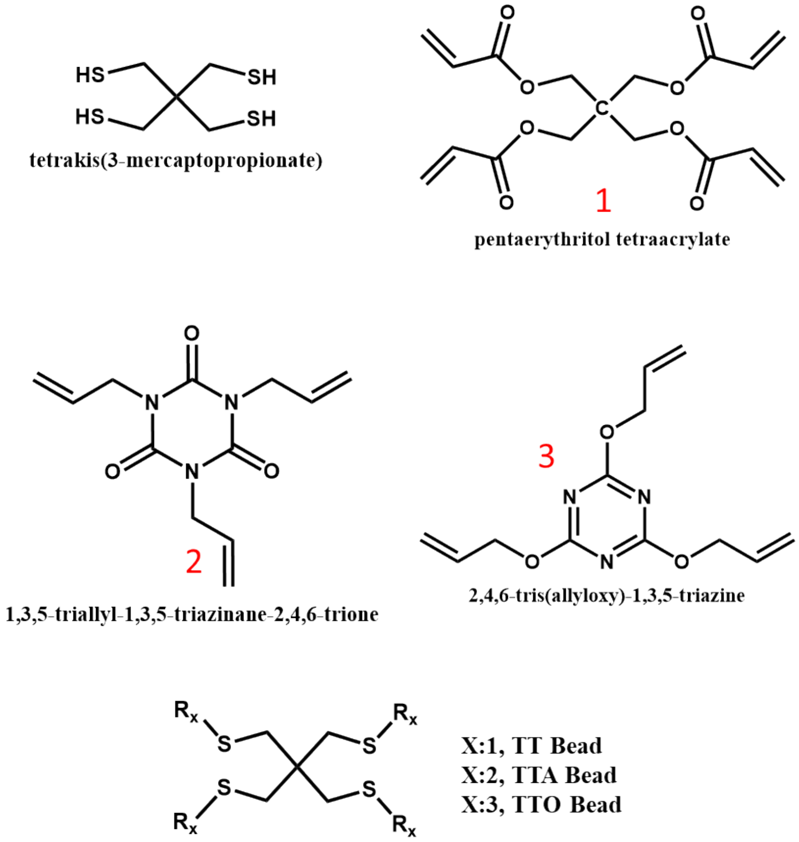

Scheme S1. Chemical structures of utilized monomers in synthesis of thiol-ene polymer beads.

Table S1. ICP-OES results of TT@CMp-vTA and CTT@CMp-vTA in regard to residual boron amount.

		Boron
1	TT@CMp-vTA	0,541 mg/g
2	CTT@CMp-vTA	0,181 mg/g
***Calib Conc. Units 0.1-1-10 mg/L**		

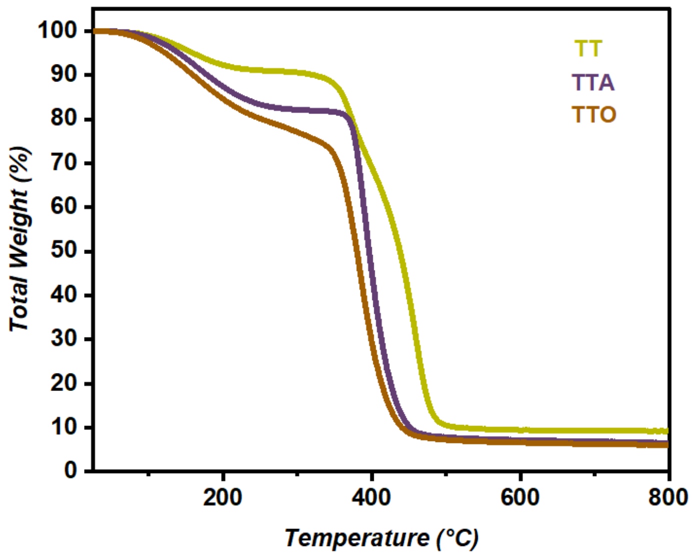

Figure S1. Thermogravimetric analysis of TT, TTA and TTO.

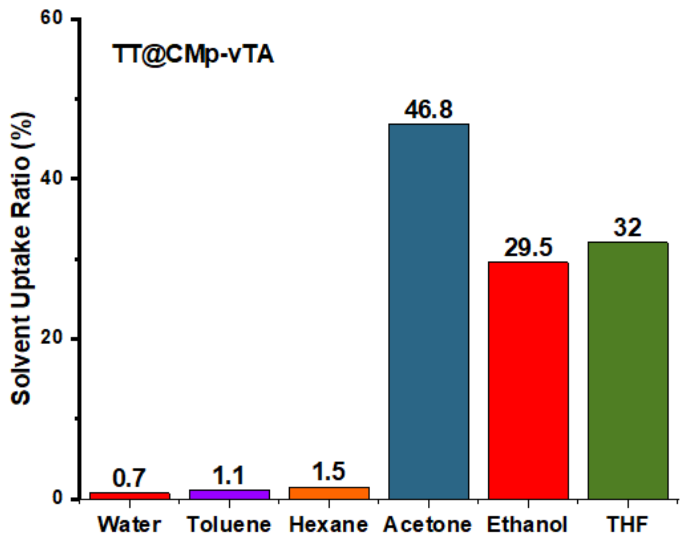

Figure S2. Solvent uptake results of TT@CMp-vTA.

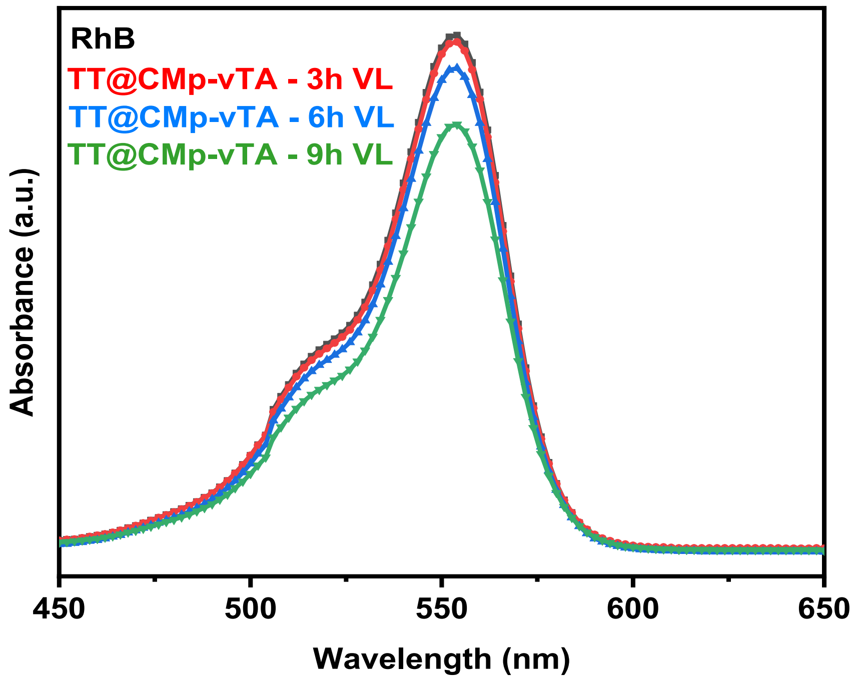

Figure S3. Visible light illumination on RhB solutions with hybrid beads (bare sample as a reference in black lines).

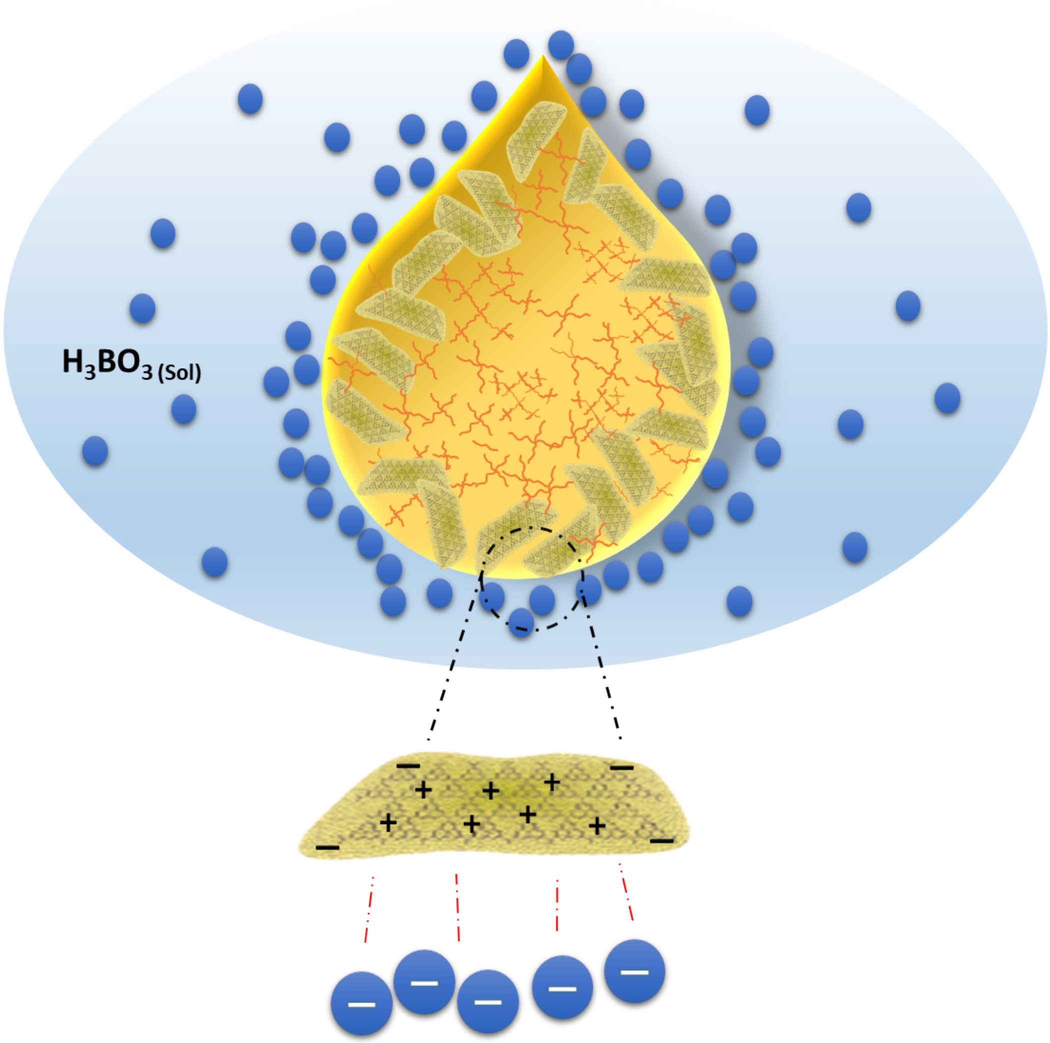

Scheme S2. Schematic depiction of interfacial interactions during liquid-liquid printing.

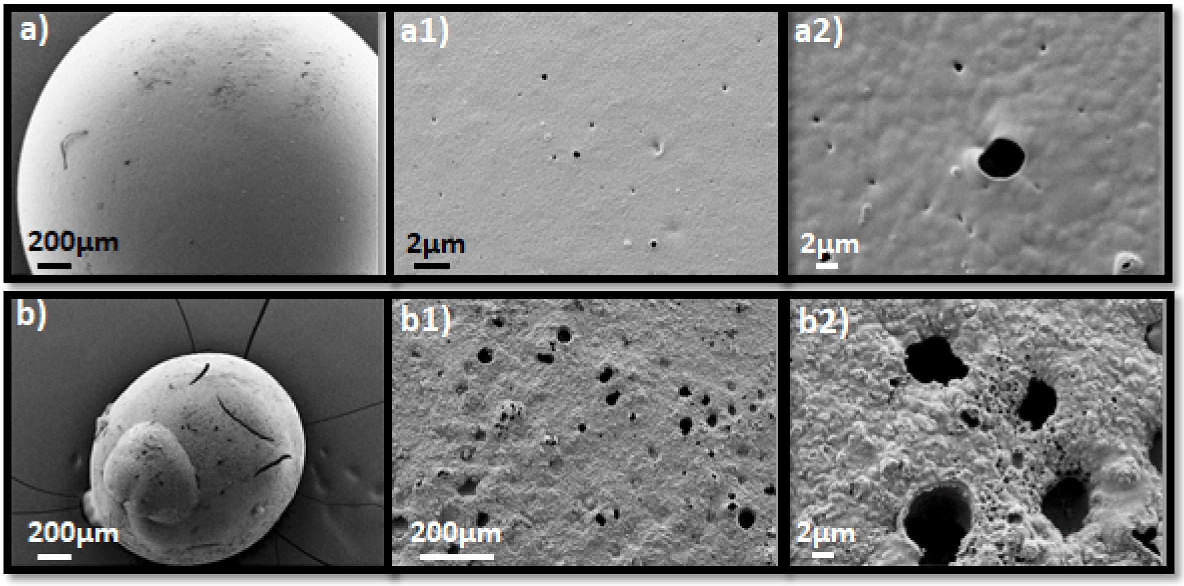

Figure S4. SEM images of TT@CMp-vTA (a1-2) and CTT@CMp-vTA (b1-2).

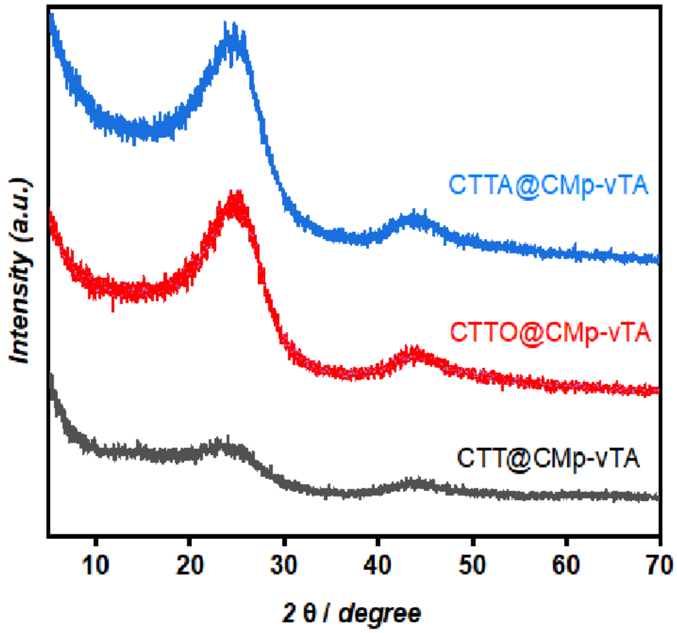

Figure S5. XRD profiles of CTT@CMp-vTA, CTTO@CMp-vTA and CTTA@CMp-vTA.

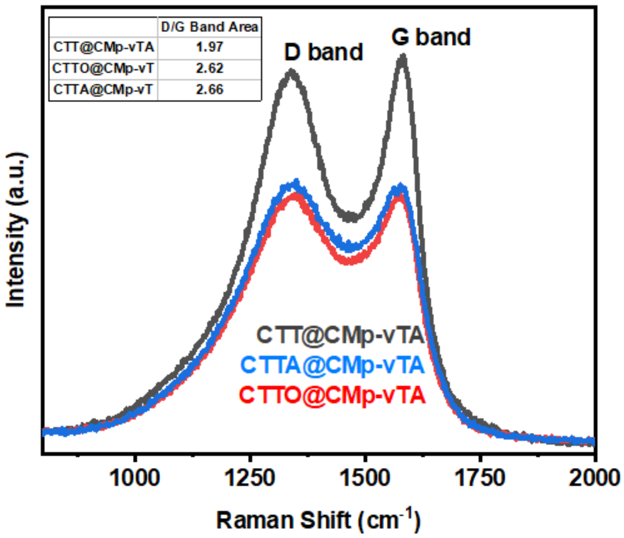

Figure S6. Raman spectra of CTT@CMp-vTA, CTTA@CMp-vTA and CTTO@CMp-vTA.

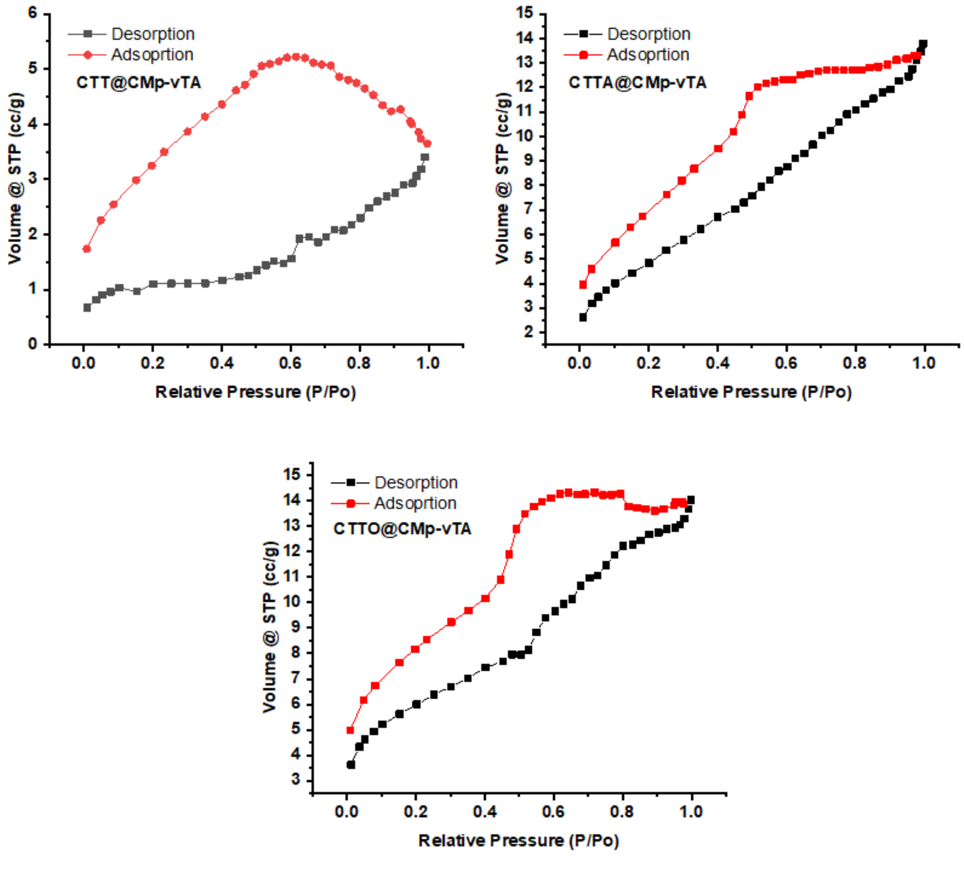

Figure S7. N_2 sorption isotherms of CTT@CMp-vTA, CTTA@CMp-vTA and CTTO@CMp-vTA, respectively.

Table S2. Chemical composition of TT, TTA, TTO, TT@CMp-vTA, TTA@CMp-vTA, TTO@CMp-vTA, CTT@CMp-vTA, CTTA@CMp-vTA and CTTO@CMp-vTA obtained by combustive elemental analysis.

	N [%]	C [%]	H [%]	S [%]
**TT**	0,04	47,2	5,53	11,91
**TT@CMp-vTA**	2,43	45,58	5,23	10,66
**CTT@CMp-vTA**	3,22	91,16	0,78	2
**TTA**	6,37	43,77	5,06	11,3
**TTA@CMp-vTA**	7,38	42,95	5,07	11,6
**CTTA@CMp-vTA**	8,68	85,98	0,9	1,45
**TTO**	6,25	41,14	4,75	11,04
**TTO@CMp-vTA**	7,62	44,4	5,18	12,16
**CTTO@CMp-vTA**	8,68	85,98	0,9	1,45
